# Faecal shedding of rotavirus vaccine in Chinese children after vaccination with Lanzhou lamb rotavirus vaccine

**DOI:** 10.1038/s41598-018-19469-w

**Published:** 2018-01-17

**Authors:** Jin-song Li, Bing Cao, Han-chun Gao, Dan-di Li, Lin Lin, LI-li LI, Na Liu, Zhao-Jun Duan

**Affiliations:** 1 National Institute for Viral Disease Control and Prevention, China CDC, Beijing, 100052 China; 2 Hunan Rucheng Rural Commercial Bank CO., Ltd, Hunan, 424104 China

## Abstract

Lanzhou lamb rotavirus vaccine (LLR) is an oral live attenuated vaccine first licensed in China in 2000. To date, > 60 million doses of LLR have been distributed to children. However, very little is known about faecal shedding of LLR in children. Therefore, faecal samples (n = 1,184) were collected from 114 children for 15 days post-vaccination in September–November 2011/2012. Faecal shedding and viral loads were determined by an enzyme immunoassay kit (EIA) and real-time RT-PCR. The complete genome was sequenced and the vaccine strain was isolated by culture in MA104 cells. Approximately 14.0% (16/114) of children had rotavirus-positive samples by EIA for at least 1 day post-vaccination. Viral loads in EIA-positive samples ranged from < 1.0 × 10^3^ to 1.9 × 10^8^ copies/g. Faecal shedding occurred as early as post-vaccination day 2 and as late as post-vaccination day 13 and peaked on post-vaccination day 5–10. One LLR strain was isolated by culture in MA104 cells. Sequence analysis showed 99% identity with LLR prototype strain. Faecal shedding of LLR in stool is common within 15 days of LLR vaccination, indicating vaccine strains can replicate in human enteric tissues.

## Introduction

Rotavirus (RV), a member of the *Reoviridae* family, has a double-stranded RNA genome with 11 segments. RV group A is recognised as the most common pathogen of acute gastroenteritis, especially severe cases, in children < 5 years of age worldwide^1^. The estimated number of RV deaths in children <5 years of age was 215,000 (range, 197,000–233,000), which accounted for 37.3% (95% confidence interval, 34.2–40.5%) of diarrhoea deaths in 2013^2^. In China, RV-associated hospitalisations account for 32–50% of all hospitalisations for diarrhoea among infants and children < 5 years of age^3^.

In 1998, an oral live tetravalent rhesus-human reassortant RV vaccine (RotaShield, Wyeth Laboratories, Marietta, PA, USA) was licensed and subsequently withdrawn from the United States (US) market due to increased risk of intussusception in infants^4^. Recently, a pentavalent bovine-human reassortant RV vaccine (PRV; RotaTeq, Merck, Whitehouse Station, NJ, USA) and a human attenuated monovalent RV vaccine (HRV; Rotarix, GlaxoSmithKline, Brentford, UK) have been licensed in many countries. Studies reported that these three vaccines caused viral shedding in stool samples within a relatively short period (i.e., 30 days) post-vaccination and also confirmed the risk of vaccine strain transmission^5,6^. Vaccine strain transmission from vaccinated children to unvaccinated contacts may lead to herd immunity. However, it also carries the risk of vaccine-derived disease in immunocompromised patients^7–9^.

A RV vaccine, Lanzhou lamb rotavirus (LLR) vaccine, has been developed and licensed in China since 2000^10^. LLR is a monovalent ovine attenuated vaccine (serotype (G10P[15])^11^ group A) that is produced in neonatal calf kidney cells. At the end of 2014, a total of 60 million doses of LLR had been distributed to children in China. LLR was shown to confer a certain level of protection against RV gastroenteritis by a population-based active surveillance study, even under a less ideal immunisation schedule^10^. However,very little is known about the post-marketing effectiveness of LLR, especially faecal shedding of LLR. Therefore, in this study, we characterised faecal shedding of LLR in infants post-vaccination.

## Results

### General patient information

A total of 120 infants (54 girls and 66 boys) aged 6–36 months, the median age is 14 months, were enrolled, including 40 children in 2011 and 80 children in 2012. Samples were unavailable in 5.0% (6/120) of children in this study. Of children with available samples, 93.0% (106/114) received their first dose and 7.0% (8/114) just received the second dose of LRR vaccine.

A total of 1,184 stool samples were collected from 114 children, including 343 samples collected in 2011 and 841 samples collected in 2012. Approximately 3.5% (4/114) of children were sampled for 4 days, 11.4% (13/114) were sampled for 5–7 days, 4.39% (5/114) were sampled for 8–9 days, and 80.7% (92/114) were sampled for 10–15 days. Concerning number of samples, 5.5% (7/114) of children had <5 samples, 29.8% (34/114) had 6–10 samples, and 64.0% (73/114) had 11–15 samples. Two or three samples per day were collected from nine children who had diarrhoea.

### RV antigen testing

Approximately 14.0% (16/114) of children had RV-positive stool samples by EIA for at least 1 day within the 15-day post-vaccination period. Faecal shedding of LLR occurred as early as post-vaccination day 2 and as late as post-vaccination day 13 and peaked on post-vaccination day 5–10 (Table 1). The mean duration ( ± standard deviation) of faecal shedding of LLR was 3.7 ± 1.6 days.

**Table 1:** Fecal shedding of LLR by post-vaccination day.

	Day post-vaccination														
	day 1	day 2	day 3	day 4	day 5	day 6	day 7	day 8	day 9	day 10	day 11	day 12	day 13	day 14	day 15
sample returned	65	98	99	91	99	97	93	82	85	72	81	71	73	64	14
EIA positive for rotavirus antigen	0	1	1	2	7	8	10	7	6	7	3	1	2	0	0
real-time PCR positive for vaccine virus	0	1	1	2	7	8	10	7	6	7	0	1	1	0	0
% positive per day(EIA)	0.00%	1.02%	1.01%	2.20%	7.07%	8.25%	10.80%	8.54%	7.06%	9.72%	3.70%	1.41%	2.82%	0.00%	0.00%
% positive per day(real-time PCR)	0.00%	1.02%	1.01%	2.20%	7.07%	8.25%	10.80%	8.54%	7.06%	9.72%	0.00%	1.41%	1.41%	0.00%	0.00%

**Figure 1:**
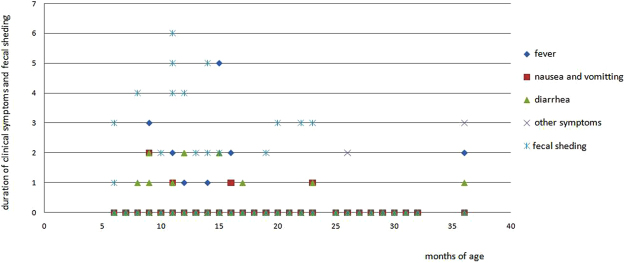
Faecal shedding of LLR vaccine. Faecal shedding occurred from 1 to 7 days post-vaccination, with most children shedding virus for 2–4 days post-vaccination. Adverse events were most surveyed between 8 to 16 months old. Most of the adverse events with courses from 1 to 2 days presented as fever, nausea and vomitting and diarrhea.

Fifty-five samples were RV-positive by EIA. Of these samples, 58.2% (32/55) had OD values ranging 0.2–0.5, 30.9% (17/55) had OD values ranging 0.5–1.0, 7.3% (4/55) had OD values ranging 1.0–1.5, and 3.6% (2/55) had OD values > 1.5. Approximately 13.2% (14/106) of children who received the first dose and 25.0% (2/8) of children who received the two doses of LLR exhibited faecal shedding of LLR, but there was no difference in vaccine viral faecal shedding between the two groups(p > 0.05, Chi-square test with continuity correction). 75%(12/16) faecal shedding of LLR were most detected in 6 to 15 months, but there was no difference in vaccine viral faecal shedding between the three age groups(1–12months,13–24 months and 25–36 months)(p > 0.05, Wilcoxon Scores test). The details of faecal shedding of LLR are presented in Fig. 1 and Table 2.

**Table 2:** LLR shedding patterns and viral loads (copies per gram of stool) of infants with rotavirus positive stool specimens

Infant	day 2		day 3		day 4		day 5		day 6		day 7		day 8		day 9		day 10		day 11		day 12		day 13	
	EIA	PCR*	EIA	PCR*	EIA	PCR*	EIA	PCR*	EIA	PCR*	EIA	PCR*	EIA	PCR*	EIA	PCR*	EIA	PCR*	EIA	PCR*	EIA	PCR*	EIA	PCR*
1	—		—		—		+ +	2.11 × 10^4^	N		—		+ +	1.3 × 10^4^	+	< 1.0 × 10^3^	+	< 1.0 × 10^3^	N		—		N	
2	—		—		—		+		++	1.7 × 10^4^	+ + + + ^#^	1.78 × 10^8^	+ +	1.1 × 10^7^	N		+	< 1.0 × 10^3^	N		—		N	
3	—		—		—		—		—		+ +	3.7 × 10^5^	—		N		+	< 1.0 × 10^3^	—		N		—	
4	—		N		—		+ +	4.9 × 10^4^	—		—		—		—		—		—		—		—	
5	N		—		—		—		—		+	< 1.0 × 10^3^	N		+ +	4.7 × 10^4^	N		—		N		—	
6	—		—		N		+	< 1.0 × 10^3^	N		N		+ +	2.3 × 10^4^	N		+	< 1.0 × 10^3^	N		N		—	
7	N		—		—		+	< 1.0 × 10^3^	+	< 1.0 × 10^3^	+ +	1.9 × 10^5^	+ + + ^#^	1.8 × 10^6^	+ +	1.7 × 10^5^	+	< 1.0 × 10^3^	+	—	—		—	
8	—		N		—		N		+	< 1.0 × 10^3^	+ +	3.9 × 10^5^	N		N		+	< 1.0 × 10^3^	N		—		—	
9	—		N		—		N		—		+	< 1.0 × 10^3^	+ + + ^#^	3.1 × 10^6^	—		N		+	—	+	—	N	
10	N		—		—		—		+	< 1.0 × 10^3^	+	< 1.0 × 10^3^	+	4.3 × 10^5^	+	< 1.0 × 10^3^	N		0		—		—	
11	—		—		N		+	2.1 × 10^3^	+	< 1.0 × 10^3^	+	4.6 × 10^4^	+ + + + ^#^	1.9 × 10^8^	+ + +	2.4 × 10^6^	—		—		—		—	
12	—		—		—		—		+ +	1.6 × 10^4^	+ +	1.5 × 10^6^	—		—		—		—		—		—	
13	+	< 1.0 × 10^3^	+ +	2.7 × 10^4^	+	1.1 × 10^4^	—		—		—		—		—		—		—				—	
14	—		—		—		—		—		—		—		—		—		—		+	< 1.0 × 10^3^	+ +	3.7 × 10^4^
15	—		—		+	< 1.0 × 10^3^	+ + + ^#^	1.3 × 10^6^	+	5.0 × 10^5^	—		—		—		—		—		—		—	
16	N		N		—		N		+	< 1.0 × 10^3^	+	< 1.0 × 10^3^	+	2.6 × 10^4^	+ +	2.7 × 10^6^	+ +	1.3 × 10^4^	+	—	—		N	

*Is real-time PCR, # samples were cultured. The data of post vaccine day1, day14, day15 were not showing for they are all negetive.

### Viral load evaluation

No RV-positive samples were detected by real-time RT-PCR from the samples of the three children who were RV-negative by EIA. Viral loads in EIA-positive samples ranged from <1.0 × 10^3^ to 1.9 × 10^8^ copies/g by real-time RT-PCR. Approximately 3.6% (2/55) of RV-positive samples by EIA had > 1.0 × 10^8^ copies/g, 65.5% (36/55) had < 1.0 × 10^4^–1.0 × 10^8^ copies/g, 23.6% (13/55) had 1 × 10^5^–1 × 10^8^ copies/g, and 7.3% (4/55) were negative for LLR (Table 2). Total viral loads of the two bottles LLR with each of 3 ml as positive control were 1.1 × 10^6^ and 2.7 × 10^6^ copies/g, respectively.

### RV cultivation and genome analysis

Cytopathy was observed in one sample on post-vaccination day 8 from a 1-year-old boy who received 1 dose of LLR. LLR strain was also detected from the culture supernatant by RT-PCR and EIA. Furthermore, 10 partial NSP3 fragments were sequenced from 10 children and two near complete genomes of LLR strains were acquired from 2 other children, however, no predominant RV or LLR NSP3 fragments was detected from samples with the four lowest viral load subjects by RT-PCR. In each sample, gene segments were 99–100% identical to corresponding gene fragments of LLR parental strains by BLASTn and rare mutations in amino acids were also found (Table 3). Genome classification analysis showed that the LLR strain is G10-P[15]-I10-R2-C2-M2-A11-N2-T3-E2-H3.

**Table 3:** the mutation of the nucleids and amino sequnce of the near complete genome.

	2012034 mutation		2014059 mutation	
segments	NS	AA	NS	AA
VP1	1485^*^:A to G 1865:C to G 3012:C TO T	616:C TO N	no	no
VP2	no	no	1041:A to C 1257:G to A 2024 A to G	323:T to P
VP3	no	no	no	no
VP4	152:T to C 1172:A to G 1328:C to T	47:V to A 440:S to L 338:Q to R	153:T to C 1175:C to A 1133:C to T	47:Vto A
VP6	no	no	no	no
VP7	no	no	no	no
NSP1	no	no	995:A to G 1330:G to A 1497:C to G	433:R to X 489:H to X
NSP2	no	no	564:G to C 726:G to A 1000:A to G	171:E to Q
NSP3	no	no	204:A to G	no
NSP4	no	no	no	no
NSP5	no	no	no	no

*The numbers show the mutaion position of nucleids and amino sequences.

### Vaccine adverse events

Approximately 15.8% (19/120) of children had adverse events, however, no family members with close contact with the vaccinated child demonstrated discomfort during the 15-day post-vaccination period. All symptoms began on day 1–5 post-vaccination. No child required outpatient treatment. There were 4.2% (5/120) of children who had low-grade fever of 37.5–38.5 °C and 1.7% (2/120) of children who had fever of 39.0 °C for 1–3 days. Approximately 2.5% (3/120) and 0.83% (1/120) of children developed nausea and vomiting for 1–3 days and 5 days, respectively. Additionally, 7.5% (9/120) of children reported diarrhoea for 1–3 days with 3–5 episodes/day. One 26-month-old girl who had an allergy to tetanus toxoid vaccine developed erythema on her body for 2 days from day 4 post-vaccination. One 7-month-old boy had irritability for 1 day on day 4 post-vaccination. 94.74% (18/19) adverse events were found in the first dose in the vaccine age between 6 to 17 months (Fig. 1). There were no differences of the adverse events within the three age groups(χ^2^ = 0.359, p > 0.05). One or more samples were collected when the child reported adverse event, however, none of the samples was positive for RV by EIA or real-time RT-PCR.

## Discussion

In China, LLR is currently the only approved vaccine for RV in children. A 3-ml dose of LLR contains > 5.5 Ig CCID_50_ live virus/ml. LLR is mainly used for children aged 2 months to 3 years at a schedule of 1 dose annually before the RV epidemic season. More than 60 million doses have been administered to children <5 years of age. However, few preclinical studies, clinical trials, and post-marketing surveillance studies of the effectiveness and adverse events of LLR are available.

In the present study, LLR was detected from day 2–13 post-vaccination and faecal shedding of LLR vaccine last from 1–7 day. One LLR strain was also isolated from samples by cultivation in MA104 cells. Faecal shedding peaked at day 5–8 post-vaccination. The faecal shedding LLR most detected in the first dose between 6 to 15 months. However, additional investigation in a greater number of cases is necessary to determine whether the number of doses has an effect on viral shedding post-vaccination.

Based on a Jennerian approach, animal strains that are naturally attenuated in humans exploited for candidate rotavirus vaccines for humans, which were shown to replicate to a lower extent in humans than in their homologous^12^. In our study, Viral loads exceeded 1.0 × 10^8^ copies/g in two samples, which is greater than 1 dose of LLR (mean viral load, 5.7 × 10^6^ copies/dose), it’s also proved that LLR can replicate in human. Compared to the rotavirus vaccines on market, after the first dose of PRV or HRV, the vaccine shedding rate of infant during 28-day post-vaccination period were 43–56% tested by EIA and 94% tested by real-time RT-PCR, and the vaccine shedding duration were > 14 days in 53.3% and >30 days in 30.0% of vaccination individuals for PRV and HRV, respectively^13,14^, LLR showed a lower vaccine shedding rate and a shorter duration of shedding. Viral replication of LLR in the human intestinal tract may be limited and less than that of PRV and HRV. The ability of replication of LLR in human is lower or rotavirus antibody can inhibit the replication of LLR virus for the first dose vaccine when children had infected wild rotavirus. Further study is necessary to evaluate potential reassortant with wild type RV strains or transmission in human.

Rare mutations were found, however, it is unknown if these mutations occurred during replication in the human intestinal tract. Additionally, whether the slightly higher viral loads observed are related to these mutations is unknown. Therefore, verification of LLR parental strains should be improved and viral shedding in stool post-vaccination must be continuously monitored.

Few reports on the protective effect and safety of LLR have been published. Du *et al*. confirmed that LLR can induce CD4 + memory T cells, which is a potential indicator of immunogenicity and protection, in mice^15^. Another study demonstrated LLR effectiveness against RV-associated hospitalisation was 73.3% in Guangzhou, China^16^ while PRV and HRV vaccine effectiveness against RV-associated hospitalisation was >89% in Hong Kong, a neighbouring region^17,18^. Additionally, LLR provided a certain level of protection against RV gastroenteritis in villages located in five townships of Hebei, China^10^. The limited replication of LLR in the human intestinal tract may attribute to its effectiveness to some extent.

Viable virus from faecal shedding of PRV and HRV can result in herd immunity against RV disease in unvaccinated young children, older children, and adults, which has been confirmed in developed regions such as Europe, the US, Latin America, and Australasia^12,19,20^. However, these effects remain unclear in developing regions^21^. PRV and HRV can also cause infection in immunodeficient and immunosuppressed individuals who have close contact with vaccinated individuals via faecal-oral transmission^21–23^. Thus, LLR may also induce herd immunity in nonvaccinated children and infection risk in immunodeficient individuals who have close contact with vaccinated individuals. LLR has been in use in China since 2000, however, LLR is not included in the National Immunization Program in China. Thus, LLR vaccination coverage rates remain very low in Guangzhou and Hebei^10,16^. The rate of LLR faecal shedding and viral loads were decreased, therefore, herd immunity induced by LLR may be lower than that of the other two RV vaccines. Herd immunity and vaccine cost-effectiveness may increase in China if vaccination coverage improves. Therefore, strategies to improve LLR coverage rates, including vaccination completion rates, should be developed.

Approximately 12.7–14.9% of HRV vaccinated individuals and 27% of PRV vaccinated individuals experienced a vaccine-related adverse event of mild or moderate intensity within 30 days^5,6^. In this study, 15.8% (19/120) of vaccinated individuals reported adverse events, such as fever, nausea, vomiting, and diarrhoea. All adverse events were noted during the first dose of LLR with an adjusted adverse events rate of 17.0% (19/112). Adverse events of LLR are similar to or slightly more severe than those of HRV and PRV. Although no RV was detected in the stool samples of children with adverse events, we cannot rule out other potential infections by other pathogens that can cause an acute gastroenteritis, such as norovirus. For instance, development of Kawasaki disease was reported in a girl who received the second dose of LLR and the first dose of a freeze-dried live attenuated hepatitis A vaccine^24^. Few serious adverse events have been reported in > 60 million doses during the 16-year period since LLR was first administered in China, and the relatively weak replication capacity in the human enteric tract suggests the vaccine is safe for use in children. However, further post-vaccination surveillance of adverse events is necessary.

There are some potential limitations associated with the present study. The study population was restricted to one community with a relatively homogeneous population. Additionally, the post-vaccination period was short and may not have been sufficient to record viral shedding and adverse events. Continued post-vaccination surveillance including additional regions and longer study duration are needed to fully evaluate LLR efficacy.

The present study described faecal shedding post-vaccination with LLR, which provides a foundation to expand its use and further research.

### Nucleotide sequence accession numbers

The two complete genomes have been deposited in the GenBank database under accession numbers KY113326-KY113347. The accession numbers of other NSP3 fragments are MF125696-MF125705.

## Materials and Methods

### Study participant enrolment

Enrollment began in September and ended in November of 2011 and 2012, when the vaccine was distributed to children in a community of Beijing, China. We enrolled infants aged 2–36 months who had been deemed eligible to receive their first to third doses of LLR by community doctors.

### Sample and data collection

After providing verbal consent, primary caretakers were given a bag including five sample collection bottles and instructions on how to collect stool samples during the 15-day post-vaccination period, other collection bottles were send to the primary caretakers when samples were took to lab. Primary caretakers were also trained to swab the stool into the sample bottle at the same time. All samples were assigned a unique study number consisting of the patient number, sample number, post-vaccination day, and collection day. All samples and clinical symptoms related to vaccine adverse events within 15 days post-vaccination were collected from primary caretakers and sent to labs on the same day by IVDC staff. All samples were stored at −20 °C at IVDC. Furthermore, study team members followed up with caretakers by telephone within 15 days post-vaccination to answer any questions about LLR. All methods described in this article is followed the protocol which has been approved by the IRB committee of National Institute for Viral Disease Control and Prevention, China CDC.

### RV antigen detection and Viral load evaluation

A 10% suspension was made by dissolving stool samples in phosphate-buffered saline for enzyme immunoassay (EIA) (Oxoid Prospect ELISA Kit, Basingstoke, United Kingdom) according to the manufacturer’s protocol. The optical density (OD) of the positive control was 2.5 while OD of the negative control was 0.1. Thus, the positive threshold for EIA was determined as 0.21.

RV double-stranded RNA (dsRNA) from 10% stool suspensions was extracted using an RNA kit (Qiagen, Valencia, USA) according to the manufacturer’s instructions. All samples from random selected three children with RV-negative samples by EIA were sent for viral load determination by real-time RT-PCR targeting the NSP3 gene of the majority of genotype of rotavirus to confirm the sensitivity of EIA. Viral loads of RV-positive samples by EIA and two bottles of LLR were also determined by the real-time RT-PCR assay. Samples when the children were uncomfortable were also detected by the real-time RT-PCR assay. Serial dilutions of purified RNA transcribed from plasmids containing the synthesised NSP3 gene were used as a quantification standard^25^. Samples with the five highest viral loads were used for virus isolation and samples with the two highest viral loads were sent for genotyping and genome sequencing. Samples with the highest viral loads from one subject were amplified by RT-PCR to confirm the virus strains with specific primers NSP3r1 and LLR NSP3R.Specific primers NSP3–1F/NSP3-1R were used to amplify a short fragment of the NSP3 region of LLR from the highest viral load samples by real-time PCR but negative by NSP3r1 and LLR NSP3R primers,then G typing for the dominant rotavirus in China, including G1,G2,G3,G4,G8 and G9 genotype rotavirus, was performed using a semi-nested PCR to genotype the samples by primers NSP3-1F/NSP3-1R^26^.

### Nucleotide sequencing and analysis

Fragments amplified by primer pairs NSP3-1F/NSP3-1R and aBT1G1,Act2G2, G3, aDT4G4,G9/VP7R were sequenced. Two complete genomes from samples with the highest viral loads were amplified and sequenced. All primers used are listed in Supplementary Table S1. Virus genotype and nucleotide sequence similarities were analysed by the RotaC v2.0 (http://rotac.regatools.be/) automated genotyping tool^27^ and BLASTn (http://www.ncbi.nlm.nih.gov/).

### Cultivation of LLR in MA104 cells

Five samples were also culture-adapted to MA104 cells as previously described with some modification^28,29^. Briefly, 1 ml of a 10% stool suspension was filtered through a 0.45-μm sterile filter (Merck Millipore, Billerica, USA) and activated in the presence of 15 μg of trypsin (Sigma‐Aldrich Cat.No. 85450 C, St. Louis, USA) for 1 h at 37 °C. Confluent monolayers of MA104 cells in roller tubes were then inoculated with stool suspensions for 1 h, unabsorbed virus was removed, and 2 ml of DMEM containing neomycin (Gibco Laboratories, California, USA) and 40 μg of trypsin were added. Tubes were harvested when cytopathic effect was complete or if no CPE is visible after 4 days, the tubes were frozen-thawed (−70 °C to room temperature) for three times before the next passage, freeze-thawed cell lysates treated with trypsin as described above for preparation of stool supernatants and was performed subsequent passages as described above. After four passages, lysates were tested for RV by real-time RT-PCR and EIA.

### Data analysis

Cumulative and daily proportions of faecal shedding of LLR for 15 days post-vaccination were calculated. Duration of shedding and peak shedding days within the 15-day post-vaccination period was determined. Adverse events related to LLR were also evaluated. The difference in vaccine viral faecal shedding between children grouped 0–12 months, 13–24 months and 25–36 months was tested by wilcoxon and the differnce in the adverse events was tested chi-square by SPSS16.0.

## Electronic supplementary material

Supplementary Information
