# Supplementary material for: Faecal shedding of rotavirus vaccine in Chinese children after vaccination with Lanzhou lamb rotavirus vaccine
Source: Sci Rep. 2018 Jan 17;8:1001. doi: 10.1038/s41598-018-19469-w (PMC5772666; doi:10.1038/s41598-018-19469-w)
Supplement: Supplementary file 1 — Supplementary Information [file 41598_2018_19469_MOESM1_ESM.doc]

**Faecal shedding of rotavirus vaccine in Chinese children after vaccination with Lanzhou lamb rotavirus vaccine**

Jin-Song Li 1^†^,Bing Cao 2, , Han-chun Gao 1,Dan-di Li 1,Lin Lin 1,LI-li LI 1,Na Liu 1^*^ and Zhao-Jun Duan1^*^

1. National Institute for Viral Disease Control and Prevention, China CDC, Beijing 100052, China.

2. HUNAN RUCHENG RURAL COMMERCIAL BANK CO., LTD

*Corresponding authors. Mailing address National Institute for Viral Disease Control and Prevention, China CDC, 100 Ying-Xin St., Xuan-Wu District, Beijing 100052, China. Tel: 86-10-8351-7145; Fax: 86-10-8354-8065; E-mail: [zhaojund@126.com](mailto:zhaojund@126.com)

Jin-song Li, lionalex@126.com; Bing Cao,[365774850@qq.com](mailto:邮箱365774850@qq.com);Han-Chun Gao,[gaohanchun@126.com](mailto:gaohanchun@126.com); Dan-Di Li, [dandili@126.com](mailto:dandili@126.com); Lin Lin,[y653@163.com;Lili](mailto:y653@163.com;Lili) Li, lilili0304@163.com; Na Liu, unali@163.com; Zhao-Jun Duan,zhaojund@126.com

S1 the primers for tne LLR complete genome and genotyping the predominant rotavirus

| prime name | primer sequence |
| --- | --- |
| LLR VP1F1 | GGCTATTAAAGCTATACAATGG |
| LLR VP1F1-r | TTCACGGTCGAAGCTACTATAG |
| LLR VP1R1-F | AGAATATACTGATTCATTAATG |
| LLRVP1R1 | AATCAACCAACCATTCCTGTATC |
| LLR VP1F2 | GTACGAGCTATAGTACCGGATC |
| LLR VP1R2 | GCTATTGAATTAGCTGCTTTCG |
| LLR VP1F3 | ATACCAGATGGTAATGTTAT |
| LLR VP1R3 | ACTTTTGATACTGCATAGGTA |
| LLR VP1F4 | ACGATAAACGATATACTTAGAG |
| LLR VP1R4 | GGTCACATCTAAGCGCTCTAATC |
| LLR VP2F1 | GGCTATTAAAGGTTCAATGGCGT |
| LLR VP2F1-1 | CCTTCTGTTTCCTCATCTTGA |
| LLRVP2R1-1 | GAATTACGCAATAGATGGTA |
| LLR VP2R1 | AGATCCGGTACTACTGACCGTGC |
| LLR VP2 F2 | CTACAGGATAGGCTGAATTTGCAC |
| LLR VP2 R2 | GATCATCTGGAACTCTCGCTACAT |
| LLR VP2F3 | TATAATAACTGCAGCTAATAG |
| LLR VP2R3 | GGTCATATCTCCACAGTGGGGT |
| LLR VP3F1 | GGCTATTAAAGCAGTACCAG |
| LLR VP3R1 | AGTATGAAGGCGCTGATCCT |
| LLR VP3F2 | ATGTATTAACAACATTGTATC |
| LLR VP3R2 | TATATCCTGAGTACGAAGTAG |
| LLR VP3F3 | TGGATCAATTCGCAAATCATAT |
| LLR VP3R3 | GGTCACATCGTGACCAGTGTG |
| LLR VP4 F1 | GGCTATAAAATGGCTTCGCTCA |
| LLR VP4 R1 | ACATGTTGTATGTGCAGTTACT |
| LLR VP4 F2 | AATCAGGTGGACTAGGGTATA |
| LLR VP4 R2 | GCTGTTCCTTGATACCGCTGAT |
| LLR VP4 F3 | ATCAATGGCCACTAATGTAATG |
| LLR VP4 R3 | GGTCACAACCTCTAGACACTAC |
| LLR NSP1F1 | GGCTTTTTTTTATGAAAAGTCTTG |
| LLR NSP1R1 | GTATACAGAATTTATCATCTCTA |
| LLR NSP1F2 | GATAGACATAGTCCAACATCACTG |
| LLR NSP1R2 | GGAAACATTTTTTGCTGGCTAGG |
| LLR NSP3F | GGCTTTTAATGCTTTTCACTG |
| nsp3f1 | CAATTGCTCATATCTAGACT |
| nsp3r1 | TGAACGTGGTGAGGTTGAAGT |
| LLR NSP3R | GGTCACATAACGCCCCTATAG |
| LLRNSP2F | GGCTTTTAAAGCGTCTCAGTC |
| nsp2f1 | ATACGTCAGCTTCCACATCG |
| nsp2r1 | GAGTTACTACTTAAATCAGTTC |
| LLRNSP2R | GGTCACATAAGCGCTTTCAAT |
| LLRNSP4F | GGCTTTTAAAAGTTCTGTTCC |
| LLRNSP4R | GGTCACATTAAGACCGTTCCT |
| vp6f1-1 | CGAAGTCTTCAACATGGATGTC |
| LLRVP6R1 | GTTGAATCAGATCACTTACAT |
| LLRVP6F2 | AGTCTTCAACATGGATGTCC |
| LLRVP6R2 | CTGGAATGACAATCTAATTGTG |
| LLRVP6F3 | TAATCCAGTAATACTGAGAC |
| LLRVP6R3 | GGTCACATCCTCTCACTACAC |
| LLR VP7 F | GGCTTTAAAAGCGAGAAT |
| llrvp7fa | CATCGGGTTACACAGCCATTC |
| LLRVP7Ra | ATATAGTTCTAATGCGATATA |
| LLR VP7 R | GGTCTCATCATACAACTCTAATC |
| LLR NSP5-6 F | GGCTTTAAAAGCGCTATAGTG |
| LLR NSP5-6 R | TCACAAAACGGGAGTGGGGAGCT |
| NSP3-1F | TGAAGCTGCAGTTGTTGCTG |
| NSP3-1R | CAYTTAATTATTGATGATGA |
| NSP3-2F | CAATATGATTATAATGAAGT |
| NSP3-2R | GATCAATTCCTTTCGATGAC |
| VP7 F | ATGTATGGTATTGAATATACCAC |
| VP7 R | AACTTGCCACCATTTTTTCC |
| aBT1G1 | CAAGTACTCAAATCAATGATGG |
| Act2G2 | CAATGATATTAACACATTTTCTGTG |
| G3 | ACGAACTCAACACGAGAGG |
| Adt4G4 | CGTTTCTGGTGAGGAGTTG |
| G9 | CTTGATGTGACTAYAAATAC |
